# Scorpion Venom Heat-Resistant Peptide Attenuates Microglia Activation and Neuroinflammation

**DOI:** 10.3389/fphar.2021.704715

**Published:** 2021-10-04

**Authors:** Xue-Fei Wu, Chun Li, Guang Yang, Ying-Zi Wang, Yan Peng, Dan-Dan Zhu, Ao-Ran Sui, Qiong Wu, Qi-Fa Li, Bin Wang, Na Li, Yue Zhang, Bi-Ying Ge, Jie Zhao, Shao Li

**Affiliations:** ^1^ Liaoning Provincial Key Laboratory of Cerebral Diseases, Department of Physiology, College of Basic Medical Sciences, Dalian Medical University, Dalian, China; ^2^ National-Local Joint Engineering Research Center for Drug-Research and Development (R&D) of Neurodegenerative Diseases, Dalian Medical University, Dalian, China; ^3^ Reproductive Medicine Centre, Affiliated Zhongshan Hospital of Dalian University, Dalian, China; ^4^ Department of Thoracic Surgery, Tongji Hospital, Tongji Medical College, Huazhong University of Science and Technology, Wuhan, China

**Keywords:** SVHRP, anti-inflammation, microglia, NF-κB, MAPKs

## Abstract

**Background:** Intervention of neuroinflammation in central nervous system (CNS) represents a potential therapeutic strategy for a host of brain disorders. The scorpion Buthus martensii Karsch (BmK) and its venom have long been used in the Orient to treat inflammation-related diseases such as rhumatoid arthritis and chronic pain. Scorpion venom heat-resistant peptide (SVHRP), a component from BmK venom, has been shown to reduce seizure susceptibility in a rat epileptic model and protect against cerebral ischemia-reperfusion injury. As neuroinflammation has been implicated in chronic neuronal hyperexcitability, epileptogenesis and cerebral ischemia-reperfusion injury, the present study aimed to investigate whether SVHRP has anti-inflammatory property in brain.

**Methods:** An animal model of neuroinflammation induced by lipopolysacchride (LPS) injection was employed to investigate the effect of SVHRP (125 µg/kg, intraperitoneal injection) on inflammagen-induced expression of pro-inflammatory factors and microglia activation. The effect of SVHRP (2–20 μg/ml) on neuroinflammation was further investigated in primary brain cell cultures containing microglia as well as the immortalized BV_2_ microglia culture stimulated with LPS. Real-time quantitative PCR were used to measure mRNA levels of inducible nitric oxide synthase (iNOS), tumor necrosis factor-α (TNF-α), interleukin (IL)-1β and IL-6 in hippocampus of animals. Protein levels of TNF-α, iNOS, P65 subunit of nuclear factor-κB (NF-κB) and mitogen-activated protein kinases (MAPKs) were examined by ELISA or western blot. Microglia morphology in animal hippocampus or cell cultures and cellular distribution of p65 were shown by immunostaining.

**Results:** Morphological study demonstrated that activation of microglia, the main component that mediates the neuroinflammatory process, was inhibited by SVHRP in both LPS mouse and cellular model. Our results also showed dramatic increases in the expression of iNOS and TNF-α in hippocampus of LPS-injected mice, which was significantly attenuated by SVHRP treatment. *In vitro* results showed that SVHRP attenuated LPS-elicited expression of iNOS and TNF-α in different cultures without cell toxicity, which might be attributed to suppression of NF-κB and MAPK pathways by SVHRP.

**Conclusion:** Our study demonstrates that SVHRP is able to inhibit neuroinflammation and microglia activation, which may underlie the therapeutic effects of BmK-derived materials, suggesting that BmK venom could be a potential source for CNS drug development.

## Introduction

Neuroinflammation characterized by upregulation of pro-inflammatory factors may represent a common mechanism for both neurodegenerative diseases (Yuan et al., 2019) such as Alzheimer’s disease (AD) (Calsolaro and Edison, 2016) and Parkinson’s disease (PD) (Gelders et al., 2018) and mental disorders such as major depression (Miller et al., 2009). Microglia, the resident immune-competent cells in CNS, are the primary source for pro-inflammatory cytokines and implicated as pivotal mediators of neuroinflammation (Umpierre and Wu, 2020). Microglia are exquisitely sensitive to their microenvironment and able to detect even the minor disturbances in CNS homeostasis (Costa et al., 2017), transforming into a so-called activated state from the “resting” state in response to neuronal damage or immunologic challenges. Activated microglia undergo morphological change, upregulate cell-surface receptors and increase the secretion of neurotrophic factors and various proinflammatory/cytotoxic factors, to clear tissue debris and repair tissue damage, or fight against microbes (Miller et al., 2009; Umpierre and Wu, 2020). However, excessive production and accumulation of cytotoxic factors such as the pro-inflammatory cytokines and reactive oxygen and nitrogen species contribute to neuronal damage and neurodegenerative processes (Miller et al., 2009; Lima Giacobbo et al., 2019; Umpierre and Wu, 2020). Therefore, suppressing microglia overactivation and neuroinflammation is an important therapeutic strategy in the drug development for brain diseases, and microglia inhibitors derived from natural products have received much attention.

Crude drugs of plant or animal origin have been used in traditional medicine in the Orient for thousands of years. The scorpion Buthus martensii Karsch (BmK) is a very mild species widely distributed in East Asia. BmK extract and BmK venom have been commonly used in Oriental medicine to relieve chronic pain and treat epilepsy, convulsion, stroke and rheumatoid arthritis. One common feature of these diseases is inflammation, implicating that BmK-derived materials might possess anti-inflammatory property. Actually, venoms from a diverse range of venomous animals have become valuable source for drug development as they are very rich in peptide toxins with high specificity and potency for particular molecular targets (Undheim et al., 2016). Quite a few peptide toxins from BmK venom have been shown to affect ion channel functions, and have anticonvulsant, anti-tumor, and analgesic activities (Uzair et al., 2018). However, no component with anti-inflammatory property has been identified from BmK or scorpion venom (SV) of any scorpion species.

SV heat-resistant peptide (SVHRP) is a heat resistant component isolated from BmK venom in our laboratory, have been shown to inhibit sodium channels in hippocampal neurons (Zhang et al., 2007), reduce susceptibility to epileptic seizures in rats (Sun et al., 2013), promote neurogenesis in adult mice (Wang et al., 2014), attenuate glial fibrillary acidic protein (GFAP) expression (Cao et al., 2015) and protect against cerebral ischemia-reperfusion injury (Wang et al., 2020). In the present study, we demonstrated that SVHRP treatment suppressed inflammagen-induced production of proinflammatory factors and microglia activation *in vivo*. Similar results were seen in neuron-glia, mixed glia or microglia cultures stimulated by LPS. Inhibition of nuclear factor-κB (NF-κB) and mitogen-activated protein kinases (MAPKs) pathways may contribute to the anti-inflammatory effect of SVHRP.

## Materials and Methods

### Materials

Culture flasks and dishes were obtained from Nunc (Roskilde, Denmark). Media including Dulbecco’s modified Eagles medium (DMEM), N2 and B27 and fetal bovine serum (FBS) for cell cultures were purchased from Invitrogen (Carlsbad, CA, United States). Horse serum (HS) was from HyClone, Thermo Scientific, United States LPS used for cell cultures from *Escherichia coli* serotype O111:B4 was obtained from EMD Millipore Corporation (Billerica, MA, United States). The ELISA kit was obtained from KeyGEN Biotech (Nanjing, China). The Griess reagent was from Beyotime Institute of Biotechnology (Jiangsu, China). c-Jun N-terminal kinase (JNK) and phospho-JNK, p38 and phospho-p38 MAPK antibodies were from Cell Signaling Technology (Danvers, MA, United States). NOS-2 (iNOS) antibody was obtained from Santa Cruz Biotechnology. Iba-1 antibody was from WAKO (Osaka, Japan). NF-κB p65 antibody was from EMD Millipore Corporation (Billerica, MA, United States). Microtubule associated protein (MAP-2) and *β*-actin antibodies were from Abcam (Cambridge, MA, United States). ABC reagents were from Vector Laboratory (Burlingame, CA, United States). Fluorescence-labeled secondary antibodies (Alexa Fluor 488, Donkey Anti-Mouse; Alexa Fluor 594, Goat Anti-Rabbit) were from invitrogen (Carlsbad, CA, United States). 4′,6-Diamidine-2′-phenylindol-dihydrochloride (DAPI) was from Roche Diagnostics GmbH (Indianapolis, Indiana, United States). LPS for animal injection from *Escherichia coli* 055: B5 and other chemicals and biochemicals were of analytical grade and were purchased from Sigma Chemical Co. (St. Louis, MO, United States).

### Drug Preparation

SVHRP was isolated from BmK venom as described previously (Wang et al., 2014; Cao et al., 2015; Wang et al., 2020). Briefly, the crude venom from BmK was dissolved in ddH_2_O and heated at 100°C for 4 h before centrifugation. Then the supernatant was further separated by FPLC (fast protein liquid chromatograph) and fraction I (P1) was collected and used for cell or animal treatment. Result from reverse-phase HPLC demonstrated the purity of P1 (designated as SVHRP) was more than 99.5%. SVHRP was freshly prepared as a stock solution (10 μg/μl) in sterile deionized and distilled water and diluted to the desired final concentrations in the treatment medium or normal saline (NS).

### Animals

C57BL/6 mice were housed in groups of three per cage under 12 h light/12 h dark cycles in controlled environment with 45–65% relative humidity at 22 ± 2°C. All animals had free access to food and water. All procedures were in accordance with the guidelines for the proper care and use of laboratory animals of the institutional and national Committees of Animal Use and Protection. The animal protocol was approved by the animal study committees of the Dalian Medical University (Ethics committee approval permit No. L2013011).

### Establishment of Mice Model of Inflammation and Administration of SVHRP

Animal model of neuroinflammation induced by LPS (Batista et al., 2019) [5 mg/kg, intraperitoneal injection (i.p)]. C57BL/6 (BW 18–22 g) mice were injected with either SVHRP (125 µg/5 ml/kg, i. p.) or NS (5 ml/kg i. p.) for 3 days before and 1 day after LPS (LPS + SVHRP or LPS group 5 mg/kg, i. p.) or NS (SVHRP or CTRL group) injection.

### Tissue Preparation and Immunohistochemical Staining

Tissue preparation and IHC staining were performed as described by Wang et al. (2014). Mice were anesthetized with chloral hydrate and perfused with 4% paraformaldehyde solution. The brains were post-fixed and cryoprotected before serial 15 μm coronal sections were made with a cryostat (Leica CM 3050 S, Leica Microsystems AG, Wetzlar, Germany). The slices that contain ventral hippocampus were used for the IHC staining. Iba-1 primary antibody (1:1,000) was used to stain microglia in combination with biotinylated secondary antibody and ABC reagents. The bound complex was visualized by color development with diaminobenzidine (DAB). The slides were visualized with a microscopy (leica Microsystems DM400B, Wetzlar, Germany) and digitally photographed.

### Immunofluorescence Staining

Tissue preparation and IF staining were performed as described by Wang et al. (2014). Tissue slide or cultured cells fixed with 4% paraformaldehyde (20 min) and permeabilized with 0.1% Triton-x-100 in PBS (15 min), were incubated with 5% BSA solution (60 min) at room temperature and diluted primary antibodies at 4°C (overnight). After three washing steps with PBS the cells were incubated with the corresponding fluorescent secondary antibodies (1 h). Additionally, cells were stained with the nuclear dye DAPI (10 min) after p65 staining. Cells were visualized using a fluorescence microscopy (leica Microsystems DM400B, Wetzlar, Germany) for Iba-1 and Map-2 staining and laser confocal scanning microscopy (Leica TCS SP5, Wetzlar, Germany) for p65 staining.

### Cells Cultures

Mouse primary cortical neuron-glia, mixed glia and enriched microglia cultures were prepared from the brains of 1-day-old C57BL/6 mice, as described by Chen et al. (2013) with some modifications. Briefly, cortical tissues were triturated after removing the meninges and blood vessels. Cells were seeded into 24-well culture plates or flasks precoated with poly-D-lysine. Seeding medium for neuron-glia and mixed glia cultures contained DMEM supplemented with 10% FBS, 10% heat-inactivated HS, 1 g/L glucose, 2 mM L-glutamine, 50 U/ml penicillin and 50 μg/ml streptomycin. The medium for neuron-glia culture was replaced 24 h and 3 days after the initial seeding (5 × 10^5^/0.5 ml/well) with fresh maintenance medium which contained DMEM supplemented with 1% N2, 2% B27, 5% HS, 2 mM L-glutamine, 50 U/ml penicillin and 50 μg/ml streptomycin. The culture was then used for treatment on day 7. The medium for mixed glia was changed every 3 days with seeding medium. On day 12, the mixed glia culture seeded in plates was used for treatment. Microglia collected from mixed glia culture in flasks by shaking were seeded into 24-well plates and used for treatment 24 h after seeding. The immortalized murine microglia cell line BV_2_ was purchased from Cell Resource Centre of Chinese academy of medical sciences (Beijing, China). Primary cultures and BV_2_ cells were pretreated with SVHRP for 1 h before LPS challenge (100 ng/ml).

### Nitrite Assay

Cells were treated with LPS (100 ng/ml) for 24 h before the supernatants were collected. Nitrite was determined immediately by a colorimetric reaction with Griess reagent. Briefly, 50 µl of culture supernatant and 50 µl of Griess reagent were incubated in the dark at room temperature for 10 min. The absorbance at 540 nm was measured with a microplate reader (iMark, Bio-Rad Laboratories, Tokyo, Japan). The sample nitrite concentration, which is an indicator of nitric oxide production, was determined from a sodium nitrite standard curve.

### Enzyme-Linked Immunosorbent Assays

Cells were stimulated with LPS for 6 h before supernatants were collected. The levels of TNF-α in the culture medium were assessed with commercial ELISA kit according to the manufacturer’s instructions.

### RNA Isolation and Quantitative PCR

Mice were anesthetized with chloral hydrate followed by immediate decapitation. Hippocampus on both sides was collected and frozen immediately in liquid nitrogen and stored at −80°C until homogenization. Total RNA from hippocampus was isolated by the TRIzol extraction method (Invitrogen, Carlsbad, CA). Total RNA was reverse transcribed with RT Primer Mix using PrimeScript™ RT Reagent Kit (Perfect Real Time) (Takara, RR047A). Real-time PCR were conducted with the SYBR® Premix Ex Taq™ II (Takara Code: DRR081A). The primers used in real-time PCR were synthesized by Takara Biotechnology (Dalian) and listed in table 1. Real-time PCR was conducted on Rotor-Gene Q (09021296, QIAGEN Hilden, Germany) for 40 cycles. After an initial denaturation step at 95°C for 30 s, temperature cycling was initiated. Each cycle consisted of 95°C for 5 s and 60°C for 30 s. Amplification specificity was checked using melting curve following the manufacturer’s instructions. Relative gene expression was calculated by the 2^−ΔΔCT^ method (Wang et al., 2014).

### Western Blot Analysis

Total protein and cytosolic or nuclear protein were extracted from cells and equal amounts of protein samples were subjected to SDS-polyacrylamide gel electrophoresis (SDS-PAGE) and transferred onto immunoblot polyvinylidene difluoride membranes (Chemicon, Temecula, CA) for immunoblotting with different antibodies. A chemiluminescence detection system (ECL, Amersham, Berkshire, England) was used to detected the antigen-antibody complexes. The intensity of the bands was analyzed by Molecular Imager Chemic Doc XR system (Bio-Rad, Hercules, CA, United States).

### Fractionation of Nuclear and Cytoplasmic Proteins

Both the nuclear and cytoplasmic proteins from cells were extracted using a Nuclear-Cytosol Extraction Kit (Applygen Technologies; P1200; Beijing, China), following the manufacturer’s instructions.

### Image Analysis of IHC and IF Staining

Images were captured via a digital camera (LEICA DFC310FX) mounted onto a microscopy (leica Microsystems DM400B, Wetzlar, Germany). For primary cultures (*n* = 3–4), 4 marginal fields and 1 central field of the coverslips where cells growed on were selected randamly for image capture with 10X objective. Moreover, average gray scale was measured to reflect staining density. For brain slices, four representative images (2088*1,560 pixels) were taken of the hippocampus from each mouse (*n* = 3). A threshold for positive staining was determined for cell bodies and processes that were marked by Iba-1 immunostaining. Results were demonstrated as the average percent area of the positive staining for all representative images (Norden et al., 2016).

### Statistical Analysis

All the experiments were performed at least in triplicate. All data were presented as the mean ± SEM. Statistical analysis was carried out by one-way analysis of variance (ANOVA) followed by Fisher’s Least Significant Difference (LSD) test. *p* < 0.05 was considered statistically significant.

## Results

### SVHRP Inhibits Inflammagen-Induced Microglia Activation and Inflammatory Response in Hippocampus

**FIGURE 1:**
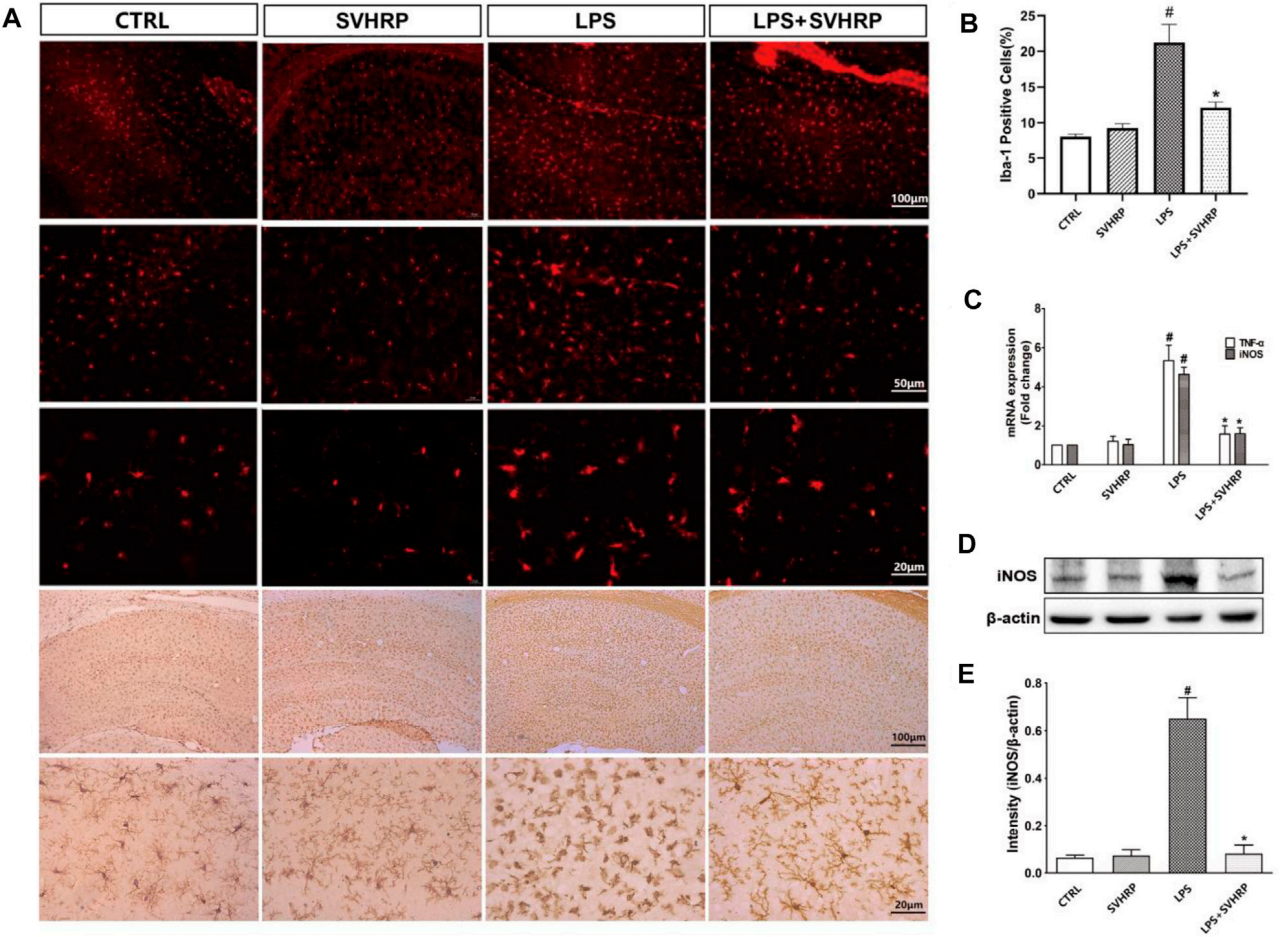
| SVHRP inhibits inflammagen-induced microglia activation and inflammatory response in hippocampus. Mice were injected with SVHRP (LPS + SVHRP or SVHRP group, 125 µg/5 ml/kg, i.p.) or NS (LPS or CTRL group, 1 ml/kg, i.p.) for 3 days before and 1 day after LPS treatment (5 mg/kg, i.p.) before the mouse brains were harvested for immuno-staining for Iba-1. **(A)** Representative images of Iba-1 staining (IF staining, upper three panels and IHC staining, lower two panels) in hippocampus were demonstrated. **(B)** The average percent area of Iba-1 positive staining of four groups was analyzed using the images from IHC staining. **(C)** mRNA expressions of TNF-α and iNOS from hippocampus were measured by real-time PCR and calculated using 2^−ΔΔCT^ method with GAPDH as the internal reference gene. The expression of iNOS protein from hippocampus was assessed by western blot. **(D)** Representative blot for iNOS and **(E)** quantification of iNOS protein normalized to β-actin. The data were expressed as the means ± SEM (*n* ≥ 3 for each group). ^#^ *p* < 0.05 compared with CTRL group, *p < 0.05 compared with LPS group.

Neuroinflammation characterized by the activation of microglia. To elucidate the effect of SVHRP on microglia activation, LPS-induced inflammation mouse model were used. We stained the coronal sections of the hippocampus of each group of mice with antibodies against Iba-1 that is a marker for microglia. Over activation of microglia cells (highly amoeboid cells and a greater number of Iba-1 positive cells) was shown in LPS model mice contrast to that in CTRL group, however, the morphology and number of microglia cells in LPS injected mice treated with SVHRP (LPS + SVHRP) were more similar to that in CTRL group (ramified cells) suggesting an inhibitory effect of SVHRP on microglia (Figures 1A,B).

It is known that inflammation is associated with upregulation of a variety of pro-inflammastory factors such as IL-1β, TNF-α, and IL-6 in hippocampus of mice injected with LPS systemically (Umpierre and Wu, 2020). In our study, mRNA levels of iNOS, TNF-α (Figure 1C) and iNOS protein level (Figures 1D,E) were dramatically increased in hippocampus of mice injected with LPS systemically in comparison with CTRL group, which can be suppressed by SVHRP treatment (LPS + SVHRP). That was, SVHRP inhibited LPS-induced upregulation of TNF-α and iNOS in hippocampus. Together, these results demonstrate that SVHRP has strong inhibitory effect on neuroinflammatory response *in vivo*.

### SVHRP Inhibits LPS-Induced Neuroinflammation *in vitro*

To examine whether SVHRP could act on brain cells directly, we applied SVHRP to CNS cell cultures and checked whether it can inhibit neuroinflammation. We conducted the investigation in three types of cultures including neuron-glia coculture (containing neurons, astrocytes and microglia), mixed glia culture (containing astrocytes and microglia) and microglia culture. LPS is an inflammagen that induce the inflammatory response through binding to the toll-like receptor four that is mainly present in microglia in CNS (Umpierre and Wu, 2020). Although LPS has no or little effect on neurons or astrocytes, neuron-glia coculture and mixed glia culture were employed here because they can better mimic the *in vivo* condition as neurons and astrocytes have crosstalk with microglia and regulate their activities (Chen et al., 2013).

**FIGURE 2:**
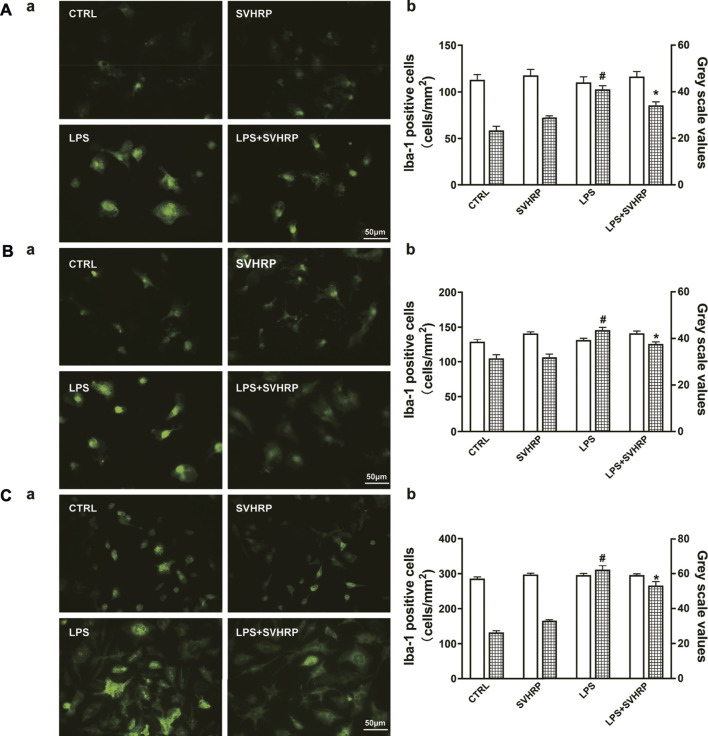
SVHRP attenuates LPS-induced upregulation of Iba-1 in microglia. IF staining for Iba-1 in primary neuron-glia **(A)**, mixed glia **(B)**, and enriched microglia **(C)** cultures were performed 24 h after LPS treatment. Cells were pretreated with vehicle or SVHRP (20 μg/ml) for 1 h before LPS challenge. **(a)** Representative images of Iba-1 positive cells (400×, Bar = 50 μm). **(b)** Cell number and average grey scale for Iba-1 staining were shown. The data were the means ± SEM (*n* ≥ 3 for each cell preparation). ^#^ *p* < 0.05 compared with CTRL group, *p < 0.05 compared with LPS group.

As decreases in pro-inflammatory factor production and Iba-1 expression could also be the consequence of toxicity of SVHRP, we examined whether SVHRP is toxic in these cultures. Our results showed that SVHRP treatment for 48 h slightly increased cell number of Iba-1 positive cells in enriched microglia (Supplementary Figure S1A) and mixed glia cultures (Supplementary Figure S1B) and cell viability in BV2 cells (Supplementary Figure S1C **)** at all doses (2–50 μg/ml) observed, but the increases were not statistically significant except for the dose of 50 μg/ml. These results suggested that SVHRP might promote the proliferation of microglia but was not toxic at doses we investigated. SVHRP was also neuroprotective in neuron-glia cocultures (Supplementary Figure S2) and neuron-enriched cultures. IF staining against Iba-1 was performed to investigate the morphological changes in microglia. The results showed that LPS treatment elicited significant increases in Iba-1 staining density, which was inhibited by SVHRP (20 μg/ml) treatment, no matter in neuron-glia (Figure 2A), mixglia (Figure 2B) or enriched microglia (Figure 2C) culture. LPS or SVHRP (20 μg/ml) had little effect on cell number of microglia (Figure 2A–Cb).

**FIGURE 3:**
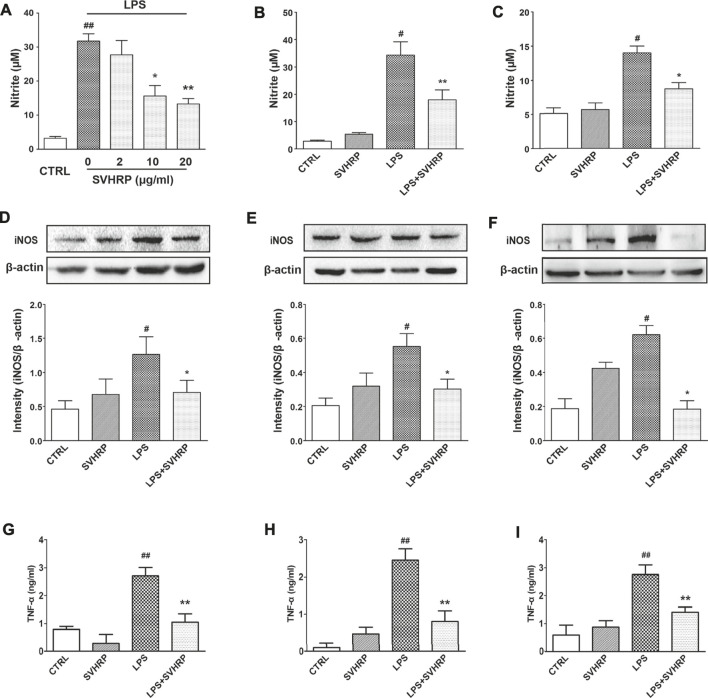
**SVHRP inhibits LPS-induced NO production, iNOS protein expression and inhibits LPS-induced TNF-**α **production *in vitro*.** Primary neuron-glia culture **(A, D)**, mixed glia culture **(B, E)** and BV_2_ cells **(C, F)** were pretreated with vehicle or SVHRP (at indicated concentrations or 20 μg/ml) for 1 h followed by LPS treatment (100 ng/ml) for 24 h. **(A–C)** Nitrite in the supernatant was measured with Griess reagent to monitor NO production. **(D–F)** Protein expression of iNOS was analyzed with western blot 24 h after LPS treatment. **(G–I)** TNF-α in the supernatant was measured with ELISA assay. The data were the means ± SEM (*n* ≥ 3 for each cell preparation). ^*#*^ *p* < *0.05*, ^##^ *p* < *0.01* compared with CTRL group, *p < *0.05*, **p < *0.01* compared with LPS group.

Additionally, results from nitrite measurement indicated that SVHRP (2–20 μg/ml) treatment dose-dependently decreased the production of NO induced by LPS in neuron-glia coculture, with more than 50% reduction at 20 μg/ml (Figure 3A). Smaller but significant inhibitions on NO production by SVHRP (20 μg/ml) in mixed glia (Figure 3B) and BV2 microglia (Figure 3C) cultures were also seen. LPS-induced upregulation in iNOS protein expression was suppressed by SVHRP (20 μg/ml) in all these cultures (Figures 3D–F), suggesting that decreased NO production by SVHRP is due to reduced level of iNOS. SVHRP alone seemed to elevate iNOS level in these cultures although the increases were not significant (Figures 3D–F). Consistent with NO production, LPS-induced TNF-α release was significantly attenuated by SVHRP (20 μg/ml) in these cultures (Figures 3G–I). In summary, these results suggest that SVHRP can inhibit LPS-induced microglia activation and neuroinflammation without non-specific toxicity.

### Inactivation of NF-κB and MAPKs May Contribute to the Anti-Inflammatory Effect of SVHRP

**FIGURE 4:**
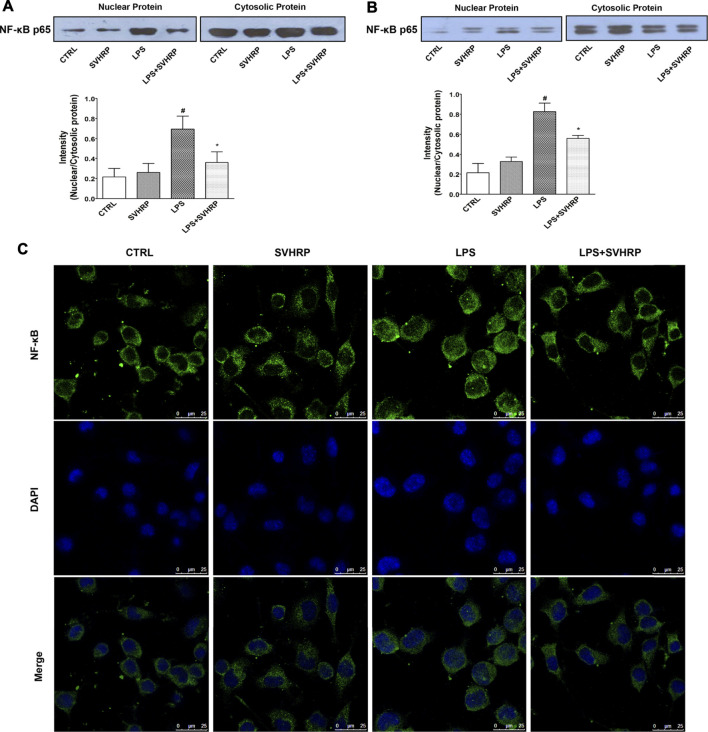
**SVHRP attenuates p65 translocation from cytoplasm to nuclei in mixed glia and BV**_**2**_ **cultures.** Mixed glia and BV_2_ cultures were pretreated with vehicle or SVHRP (20 μg/ml) for 1 h, followed by LPS treatment (100 ng/ml) for 1 h. Cytoplasmic and nuclear extracts were separated by SDS-PAGE and immunoblotted with anti-p65 antibody. Representative blots and quantification of nuclear/cytosolic ratio of p65 were shown for mixed glia culture **(A)** and BV_2_ cells **(B)**. The data were the means ± SEM (*n* ≥ 3 for each cell preparation). ^*#*^ *p* < *0.05* compared with CTRL group, *p < *0.05* compared with LPS group. **(C)** Representative image using laser confocal scanning microscopy showed subcellular localization of p65 subunit with DAPI indicating nuclei in BV_2_ cells. Bar = 25 μm.

**FIGURE 5:**
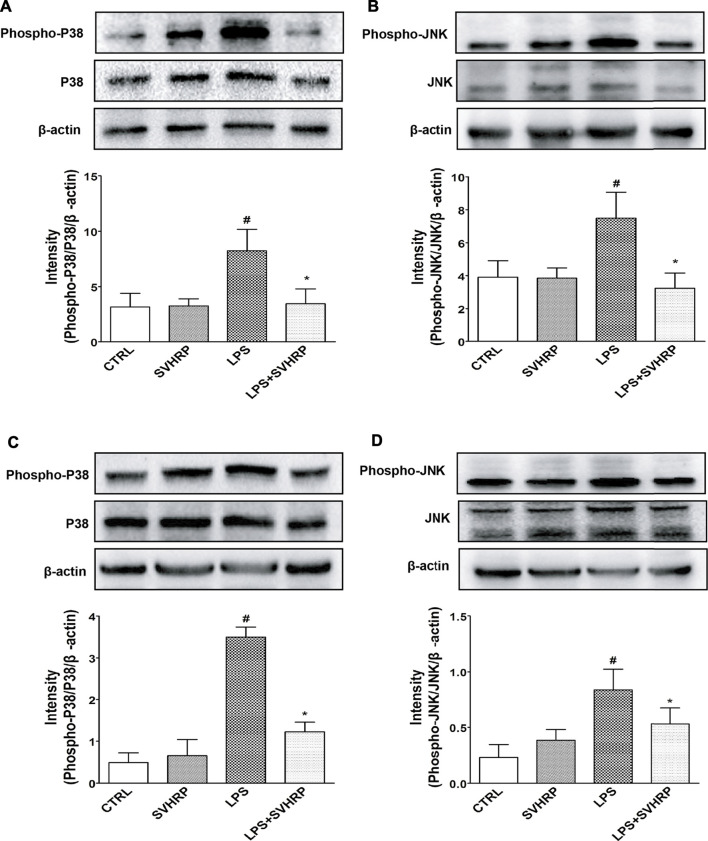
**SVHRP suppresses LPS-induced phosphorylation of p38 and JNK MAPKs in mixed glia and BV**_**2**_ **cultures.** Mixed glia and BV_2_ cultures were pretreated with vehicle or SVHRP (20 μg/ml) for 1 h, followed by LPS treatment (100 ng/ml) for 1 h. Cell lysates were subjected to western blot analysis using antibodies specific for phosphorylated forms of p38 or JNK MAPK. The relative protein levels were quantified by densitometry scanning and normalized to total p38 or JNK MAPKs and *β*-actin. Representative blotting and quantification of relative band intensities of phosphorylated p38 **(A)** or JNK **(B)** in mixed glia culture and those in BV_2_ cells **(C, D)** were shown. The data were the means ± SEM (*n* = 3). ^#^ *p* < *0.05* compared with vehicle group alone, *p < 0.05 compared with LPS group alone.

To further elucidate the mechanisms underlying the inhibition of LPS-elicited inflammatory response by SVHRP, we examined the effect of SVHRP on activation of NF-κB, which is a key transcription factor involved in the upregulation of pro-inflammatory mediators such as TNF-α and iNOS (Miller et al., 2009; Umpierre and Wu, 2020). Translocation of p65 subunit of NF-κB from cytoplasm to nucleus was evaluated by western blot or fluorescence microscopy and used as an index of NF-κB activation. Semi-quantitation of p65 protein by western blot analysis demonstrated an increased nuclear/cytosolic ratio in LPS-stimulated cells, which was significantly reduced by SVHRP in both mixed glia (Figure 4A) and BV_2_ cells (Figure 4B). The localization of p65 in BV_2_ cells was also revealed by IF staining of p65 with the nuclei visualized by DAPI staining. Consistent with the result from western blot, most of the BV_2_ cells stimulated with LPS showed translocation of p65 to the nucleus, which is significantly reduced by SVHRP (Figure 4C). Along with NF-κB, MAPKs are known to play a critical role in the signaling pathways that induce a series of proinflammatory cytokines and iNOS in glial cells (Miller et al., 2009). In mixed glia culture (Figures 5A,B) and BV_2_ cells (Figures 5C,D), LPS-elicited phosphorylation of p38 and JNK could be notably suppressed by SVHRP. These results suggest that the anti-inflammatory effect of SVHRP might be associated with the inhibition of NF-κB and MAPKs pathways.

## Discussion

In this study, we demonstrated that SVHRP, a component from BmK venom, was capable of inhibiting neuroinflammation and microglia activation both *in vivo* and *in vitro*. Microglia are sensitive to various stimuli such as neuronal death and infection. In contrast, LPS-induced neuroinflammation is the consequence of direct effect of the inflammagen on microglia (Miller et al., 2009; Umpierre and Wu, 2020). SVHRP was shown to suppress the production of pro-inflammatory factors and microglia activation in both contexts (Figures 1–3). In addition, SVHRP attenuated LPS-induced activation of NF-κB and MAPKs pathways that are critical for downstream gene expression of pro-inflammatory factors *in vitro* (Figure 4). All these effects can be seen in microglia cultures (Figures 2C, 3C,F,I, 4B,C, 5C,D), so it is definite that SVHRP can target microglia directly. The anti-inflammatory effects of SVHRP *in vivo* and in co-cultures containing microglia are probably due to the direct action on microglia, as these cells are the pivotal mediators of neuroinflammation. Although SVHRP is a component derived from toxic venom, it did not cause reduction in either cell number or viability of microglia at doses used in this study (Figures 2A–C; Supplementary Figure S1). SVHRP was also neuroprotective in neuron-glia cocultures (Supplementary Figure S2). Thus, our results suggest that the anti-inflammatory effect of SVHRP is specific and not due to cell toxicity.

Intervention of signaling pathways such as NF-κB has been an important strategy in the development of anti-inflammatory therapy (Jha et al., 2019). Inactivation of NF-κB and MAPKs pathways might be critical for the anti-inflammatory effect of SVHRP, it remains unknown, however, what receptors or ion channels mediate this action. Black et al. have reported that sodium channel activity regulates microglia function including LPS-induced inflammatory response (Hossain et al., 2018). It is likely that SVHRP, a neuronal sodium channel blocker (Zhang et al., 2007), may also act on microglial sodium channels and modulate immune function of microglia (Alrashdi et al., 2019). More evidence is needed to confirm that sodium channels are direct target for SVHRP that mediates the anti-inflammatory effect of it.

In Oriental medicine, BmK-derived materials have traditionally been used to treat chronic pain, epilepsy and rheumatoid arthritis, which are all inflammation-related diseases. However, traditional remedies that frequently use natural products are usually not supported by modern evidence-based medicine due to, at least in part, the missing knowledge of the effective components and therapeutic mechanisms of natural drugs, even that they could be therapeutically effective. Thus, great efforts have been made to isolate and purify bioactive components from crude drugs of plant or animal origin and study the cellular and molecular mechanisms of these components. Both neurons (Adams and Lewis, 2017; Zou et al., 2020) and glial cells (Choi et al., 2011) could be target cells for natural products including toxins from venomous animals and compounds from medicinal herbs. Up to now, most of the experimental studies on SV demonstrate that it causes inflammation due to envenoming by scorpions (Choi et al., 2011). Our study is the first that provide scientific evidence for anti-inflammatory effect of component from SV. This implicates that the therapeutic effect of traditional remedies using BmK-related materials might derive from the anti-inflammatory component in the venom.

## Conclusion

Our study demonstrates that SVHRP, a heat-resistant component from BmK venom, possesses anti-inflammatory property in CNS both *in vivo* and *in vitro*. Natural products such as BmK venom that has been proved to be effective in traditional medicines may serve as good sources for drug development for CNS diseases where neuroinflammation plays a critical role in the development of these diseases.

## Data Availability

The raw data supporting the conclusions of this article will be made available by the authors, without undue reservation.

## Supplementary Material

Supplementary Figure S1 SVHRP promotes the proliferation of microglia but was not toxic at doses that inhibits microglia activation. Cell number of Iba-1 positive cells in enriched microglia **(A)** and mixed glia cultures **(B)** and cell viability in BV2 cells **(C)** at all doses (2–50 μg/ml) after SVHRP treatment for 48 h. The data were the means ± SEM of at least three independent experiments. #*p* < 0.05 compared with the control cultures, **p* < 0.05 compared with LPS-treated cultures.

Supplementary Figure S2 SVHRP protects cortical neurons in neuron-glia culture against LPS-induced neurotoxicity. Primary neuron-glia culture was pretreated with vehicle or SVHRP (20 μg/ml) for 1 h before LPS challenge. Neurons in the culture were visualized by immunofluorescent staining against a neuronal marker MAP-2. Representative image of MAP-2 positive cells in low. The data were the means ± SEM of at least three independent experiments. #*p* < 0.05 compared with the control cultures, #*p* < 0.05 compared with LPS-treated cultures.

## Abbreviations

BmK, Buthus martensii Karsch; SV, scorpion venom; SVHRP, scorpion venom heat-resistant peptide; CNS, central nervous system; LPS, lipopolysacchride; NO, nitric oxide; iNOS, nitric oxide synthase; TNF-α, tumor necrosis factor-α; IL, interleukin; NF-κB, nuclear factor-κB; MAPKs, mitogen-activated protein kinases; Iba-1, Ionized calcium binding adaptor molecule-1; IHC, immunohistochemical; IF, immunofluorence.
