# Corrigendum: Scorpion Venom Heat-Resistant Peptide Attenuates Microglia Activation and Neuroinflammation

**DOI:** 10.3389/fphar.2021.791953

**Published:** 2021-11-26

**Authors:** Xue-Fei Wu, Chun Li, Guang Yang, Ying-Zi Wang, Yan Peng, Dan-Dan Zhu, Ao-Ran Sui, Qiong Wu, Qi-Fa Li, Bin Wang, Na Li, Yue Zhang, Bi-Ying Ge, Jie Zhao, Shao Li

**Affiliations:** ^1^ Liaoning Provincial Key Laboratory of Cerebral Diseases, Department of Physiology, College of Basic Medical Sciences, Dalian Medical University, Dalian, China; ^2^ National-Local Joint Engineering Research Center for Drug-Research and Development (R&D) of Neurodegenerative Diseases, Dalian Medical University, Dalian, China; ^3^ Reproductive Medicine Centre, Affiliated Zhongshan Hospital of Dalian University, Dalian, China; ^4^ Department of Thoracic Surgery, Tongji Hospital, Tongji Medical College, Huazhong University of Science and Technology, Wuhan, China

**Keywords:** SVHRP, anti-inflammation, microglia, NF-κB, MAPKs

In the original article, there was a mistake in Figure 1 and its caption as published. The label for group 4 in Figure 1A is misspelled, it is supposed to be “LPS+SVHRP” instead of “LPS+SVHRSP”. Furthermore, in the caption for Figure 1, the description for Figure 1E was missing, which is the quantification result for the immunoblotting shown in **(D)**. The correct Figure 1 and caption appear below.

**FIGURE 1:**
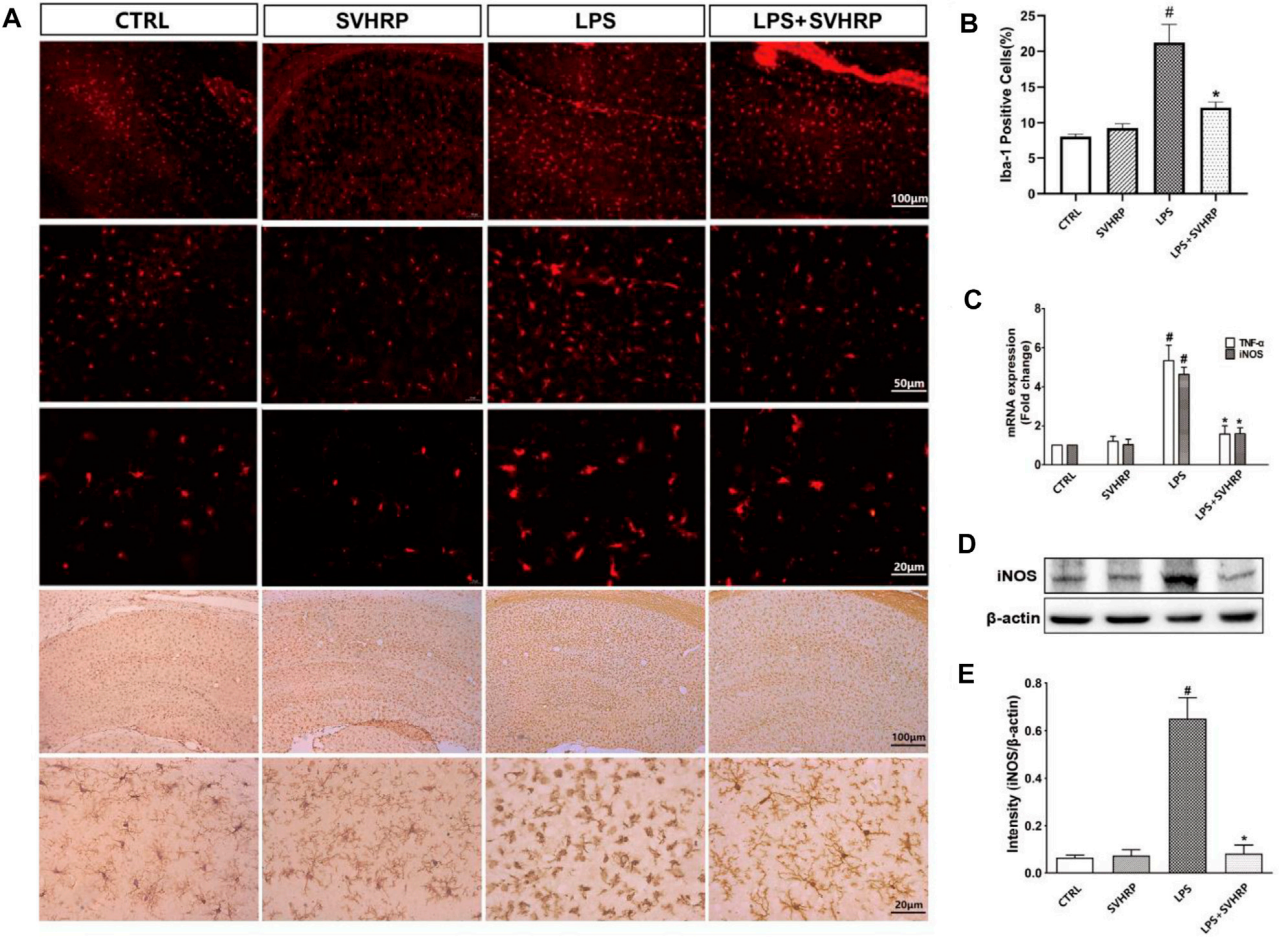
SVHRP inhibits inflammagen-induced microglia activation and inflammatory response in hippocampus. Mice were injected with SVHRP (LPS + SVHRP or SVHRP group, 125 µg/5 ml/kg, i.p.) or NS (LPS or CTRL group, 1 ml/kg, i.p.) for 3 days before and 1 day after LPS treatment (5 mg/kg, i.p.) before the mouse brains were harvested for immuno-staining for Iba-1. **(A)** Representative images of Iba-1 staining (IF staining, upper three panels and IHC staining, lower two panels) in hippocampus were demonstrated. **(B)** The average percent area of Iba-1 positive staining of four groups was analyzed using the images from IHC staining. **(C)** mRNA expressions of TNF-α and iNOS from hippocampus were measured by real-time PCR and calculated using 2^−ΔΔCT^ method with GAPDH as the internal reference gene. The expression of iNOS protein from hippocampus was assessed by western blot. **(D)** Representative blot for iNOS and **(E)** quantification of iNOS protein normalized to β-actin. The data were expressed as the means ± SEM (*n* ≥ 3 for each group). ^#^ *p* < 0.05 compared with CTRL group, *p < 0.05 compared with LPS group.

Additionally, there was a mistake in the caption for Figure 2 as published. In Figure 2, the value of the Bar in Figures 2A–C, a is supposed to be 50 instead of 20 μm. The correct Figure 2 appears below.

**FIGURE 2:**
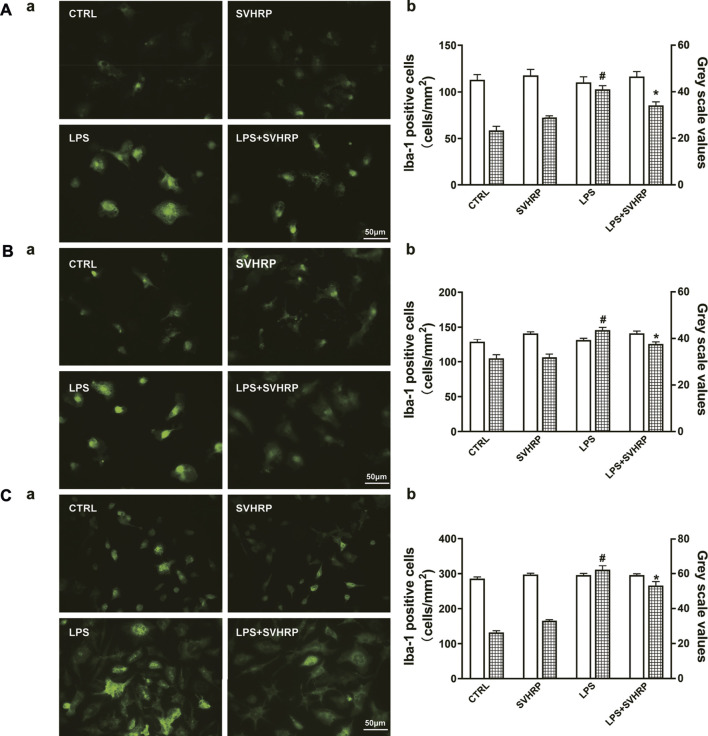
SVHRP attenuates LPS-induced upregulation of Iba-1 in microglia. IF staining for Iba-1 in primary neuron-glia **(A)**, mixed glia **(B)**, and enriched microglia **(C)** cultures were performed 24 h after LPS treatment. Cells were pretreated with vehicle or SVHRP (20 μg/ml) for 1 h before LPS challenge. **(a)** Representative images of Iba-1 positive cells (400×, Bar = 50 μm). **(b)** Cell number and average grey scale for Iba-1 staining were shown. The data were the means ± SEM (*n* ≥ 3 for each cell preparation). ^#^ *p* < 0.05 compared with CTRL group, *p < 0.05 compared with LPS group.

The authors apologize for this error and state that this does not change the scientific conclusions of the article in any way. The original article has been updated.

