# Supplementary figures and images for: Scorpion Venom Heat-Resistant Peptide Attenuates Microglia Activation and Neuroinflammation
Source: Front Pharmacol. 2021 Oct 4;12:704715. doi: 10.3389/fphar.2021.704715 (PMC8524240; doi:10.3389/fphar.2021.704715)

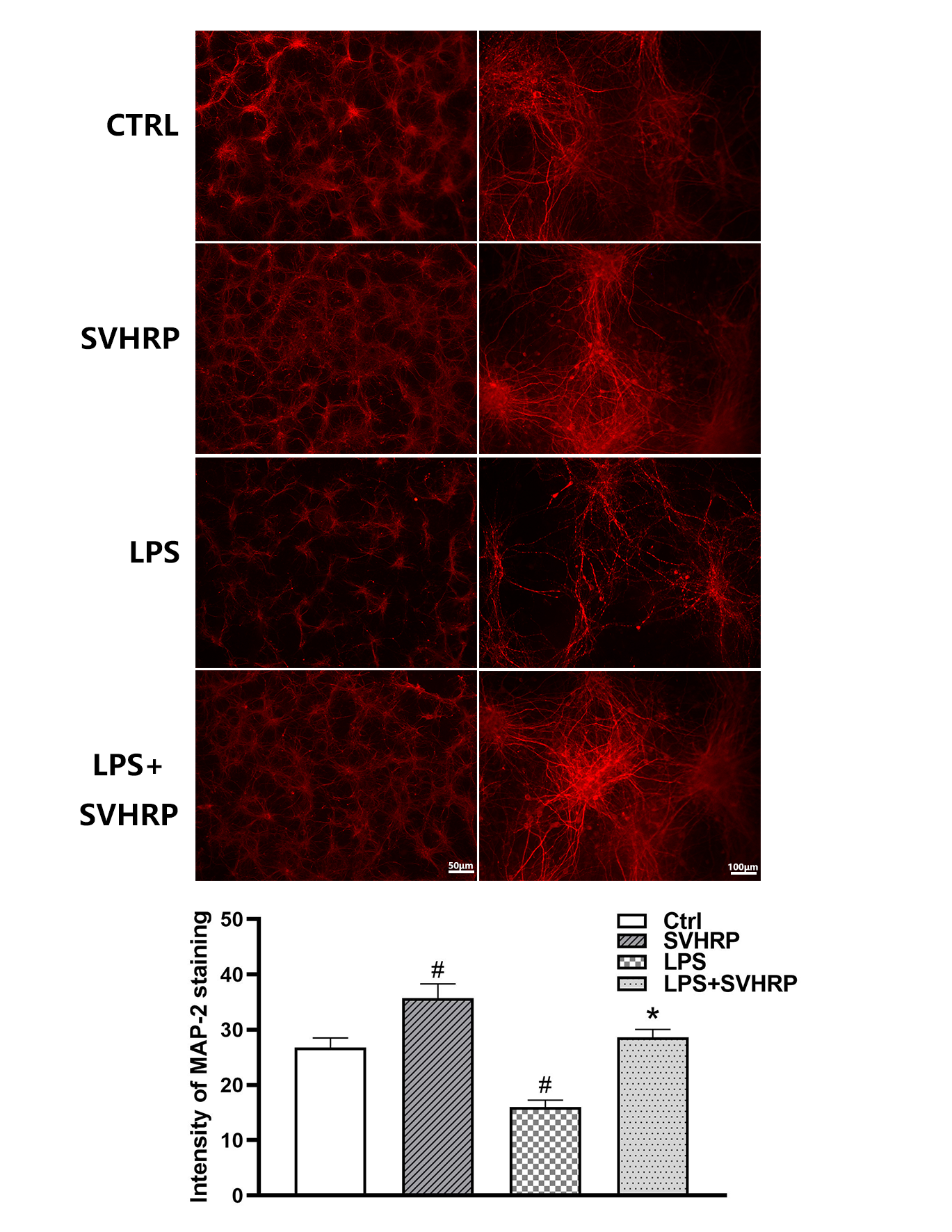

Supplement: Supplementary file 1 [file image2.tif]

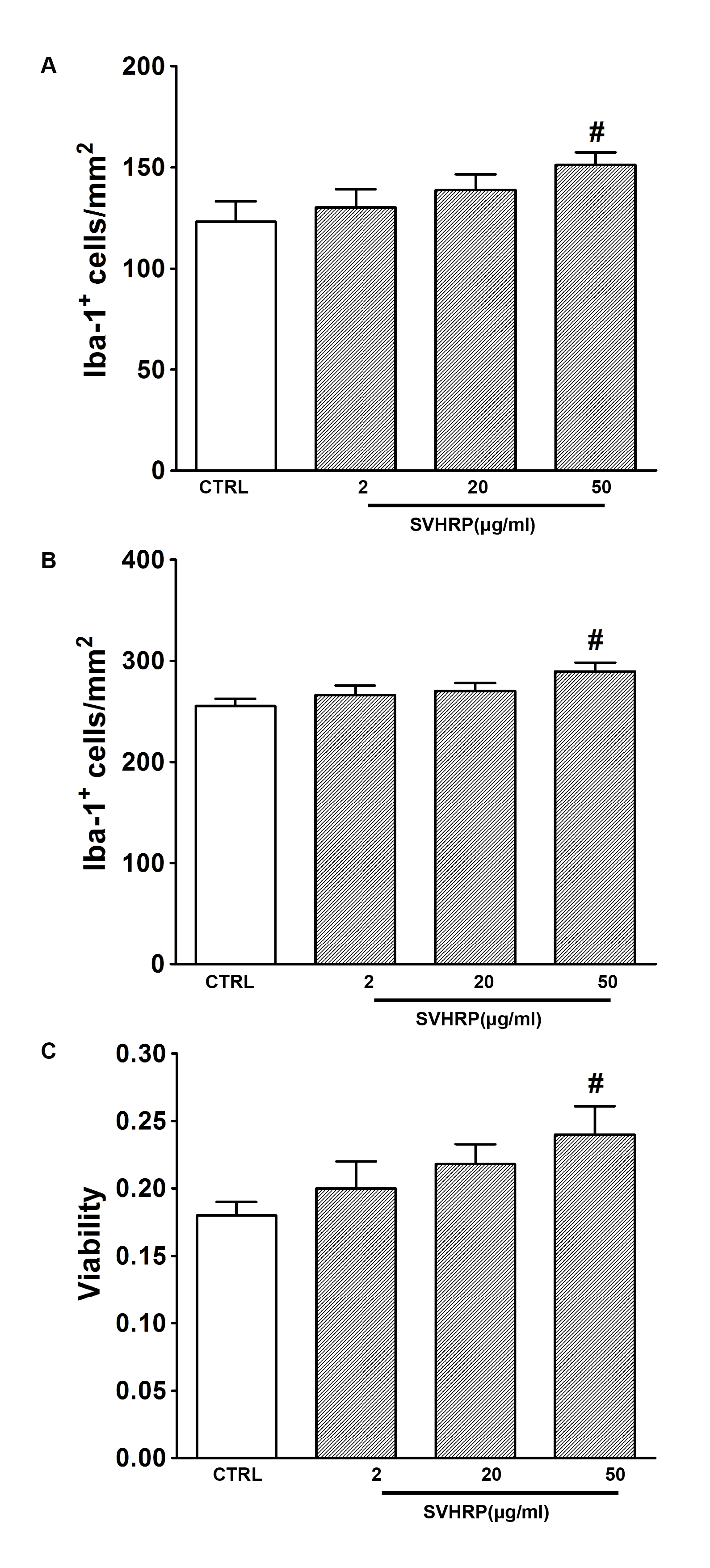

Supplement: Supplementary file 2 [file image1.tif]
